# Contribution of plasmid-encoded peptidase S8 (PrtP) to adhesion and transit in the gut of *Lactococcus lactis* IBB477 strain

**DOI:** 10.1007/s00253-017-8334-1

**Published:** 2017-05-24

**Authors:** Joanna Maria Radziwill-Bienkowska, Véronique Robert, Karolina Drabot, Florian Chain, Claire Cherbuy, Philippe Langella, Muriel Thomas, Jacek Karol Bardowski, Muriel Mercier-Bonin, Magdalena Kowalczyk

**Affiliations:** 1 Institute of Biochemistry and Biophysics, Polish Academy of Sciences, Pawinskiego 5A, 02-106 Warsaw, Poland; 2 Micalis Institute, INRA, AgroParisTech, Université Paris-Saclay, 78350 Jouy-en-Josas, France; 3 Warsaw University of Life Sciences–SGGW, Nowoursynowska 166, 02-787 Warsaw, Poland; 4 Toxalim (Research Centre in Food Toxicology) UMR INRA 1331, 180 chemin de Tournefeuille, BP 93173, 31027 Toulouse cedex 3, France

**Keywords:** *Lactococcus lactis*, Adhesion, PrtP, HT29-MTX cell line, Confocal microscopy, C57Bl/6 mice

## Abstract

The ability of *Lactococcus lactis* to adhere to the intestinal mucosa can potentially prolong the contact with the host, and therefore favour its persistence in the gut. In the present study, the contribution of plasmid-encoded factors to the adhesive and transit properties of the *L. lactis* subsp. *cremoris* IBB477 strain was investigated. Plasmid-cured derivatives as well as deletion mutants were obtained and analysed. Adhesion tests were performed using non-coated polystyrene plates, plates coated with mucin or fibronectin and mucus-secreting HT29-MTX intestinal epithelial cells. The results indicate that two plasmids, pIBB477a and b, are involved in adhesion of the IBB477 strain. One of the genes localised on plasmid pIBB477b (AJ89_14230), which encodes cell wall-associated peptidase S8 (PrtP), mediates adhesion of the IBB477 strain to bare, mucin- and fibronectin-coated polystyrene, as well as to HT29-MTX cells. Interactions between bacteria and mucus secreted by HT29-MTX cells were further investigated by fluorescent staining and confocal microscopy. Confocal images showed that IBB477 forms dense clusters embedded in secreted mucus. Finally, the ability of IBB477 strain and its ΔprtP deletion mutant to colonise the gastrointestinal tract of conventional C57Bl/6 mice was determined. Both strains were present in the gut for up to 72 h. In summary, adhesion and persistence of IBB477 were analysed by in vitro and in vivo approaches, respectively. Our studies revealed that plasmidic genes encoding cell surface proteins are more involved in the adhesion of IBB477 strain than in the ability to confer a selective advantage in the gut.

## Introduction

*Lactococcus lactis* is one of the most widely used lactic acid bacterium (LAB) in the dairy industry. It serves as a starter culture for the production of a variety of cheeses, as well as other dairy products such as sour cream and buttermilk. Owing to the long history of safe consumption and the availability of molecular tools, lactococci have a great potential as mucosal delivery vehicles for therapeutic and prophylactic molecules (Bermudez-Humaran et al. 2011; Hugentobler et al. 2012; del Carmen et al. 2013; Szatraj et al. 2014; Kasarełło et al. 2016). The ability of *L. lactis* to adhere to the intestinal mucosa can potentially prolong the contact with the host, and therefore favour its persistence in the gut. After consumption, *L. lactis* is confronted to the digestive tract harsh conditions, and in this context, we tested if the contribution of the adhesion-mediating factors could confer a selective advantage in the gut.

*L. lactis* strains characteristically contain many plasmids that vary in size and copy number. These plasmids encode numerous traits of biotechnological significance, including lactose and casein utilisation, flavour development, stress response, bacteriophage resistance and production of bacteriocins (for review, see Ainsworth et al. 2014). Recent studies indicate that some of the genes localised on lactococcal plasmids are potentially involved in adhesion of *L. lactis* to the intestinal mucosa. The *prtP* gene from pWV05 plasmid of the *L. lactis* Wg2 strain, coding for the cell wall-anchored proteinase, was shown to enhance cell hydrophobicity and adhesion to solid surfaces (Habimana et al. 2007). Two genes localised on the pKP1 plasmid of *L. lactis* BGKP1 strain, *aggL* and *mbpL*, encoding aggregation and mucin-binding proteins, were reported to mediate adhesion to HT29-MTX cells and colonic mucus, respectively (Lukić et al. 2012). Two plasmid genes with key roles in adhesion were also identified in *L. lactis* TIL448 strain, *yhgE2* gene coding for backbone pilin, which was shown to be involved in adhesion of TIL448 to Caco-2 cell line, and *muc* gene coding for mucus-binding protein (Meyrand et al. 2013). Both genes were found to contribute to the ability of TIL448 strain to adhere to pig gastric mucin (PGM) under static and dynamic conditions (Le et al. 2013). For *L. lactis* subsp. *cremoris* IBB477, the model strain in the present study, eight proteins out of 63 predicted by PSORTb as extracellular or cell wall attached are localised on its plasmids (Radziwill-Bienkowska et al. 2016). Taking into account that cell surface-associated macromolecules are considered to play an important role in the adhesion of LAB to the gastrointestinal tract (GIT), the nature and functional role of plasmidic genes in IBB477 adhesive phenotype remain to be unravelled.

Bacteria can attach to different components of the intestinal mucosa, in particular mucins and proteins of the extracellular matrix (ECM), such as laminin, collagen and fibronectin (Vélez et al. 2007). A well-established in vitro model to study bacterial adhesion is the mucus-secreting HT29-MTX cell line, a homogenous subpopulation of goblet cells selected from a mostly undifferentiated human colon carcinoma HT29 cell population after growth adaptation to anti-cancer drug methotrexate (MTX) (Lesuffleur et al. 1990). Owing to its mucus-secreting phenotype, the HT29-MTX cell line is widely used to investigate adhesive properties of bacteria (Coconnier et al. 1992; Gopal et al. 2001; de los Reyes-Gavilán et al. 2011; Turpin et al. 2012; Kebouchi et al. 2016).

Adhesion may confer a selective advantage for transient food-borne bacteria to persist within the GIT, even though consistent experimental evidence is still lacking. In the studies reported by Turpin et al. (2012, 2013), three lactobacilli, namely *Lactobacillus fermentum*, *Lactobacillus paraplantarum* and *Lactobacillus salivarius*, displayed equivalent adhesive properties to HT29-MTX cells and to their non-mucus-secreting counterparts HT29 (Turpin et al. 2012), even though in gnotobiotic rats inoculated with the same bacteria as a cocktail, their colonisation profiles were clearly different (Turpin et al. 2013). Regarding *L. lactis*, its ability to survive the gastrointestinal transit is well documented (Klijn et al. 1995; Drouault et al. 1999; Kimoto et al. 2003). In a very recent study on the CNCM I-1631 strain, its longer persistence in a subgroup of rats exhibiting a “permissive” phenotype of microbiota was possibly attributed to its ability to adhere to the intestinal mucosa (Zhang et al. 2016). Indeed, deletion of *srtA* gene encoding housekeeping sortase A resulted in a shorter persistence of the CNCM I-1631 strain in the permissive rat subgroup. However, when introduced in rodents associated with single or multiple bacterial strains, the ability of *L. lactis* to persist in the gut varied between the studies (Schlundt et al. 1994; Brockmann et al. 1996; Alpert et al. 2003; Boguslawska et al. 2009; McNulty et al. 2011). In the GIT of germ-free animals, like in mice, *L. lactis* is able to efficiently colonise and persist when inoculated as a single strain (Gruzza et al. 1992; Corthier et al. 1998; Roy et al. 2008). Moreover, some particular strains, such as *L. lactis* IBB477 and *L. lactis* CNCM I-1631, are able to persist in the GIT of gnotobiotic animals for the entire monitoring period (at least 2 weeks) (Boguslawska et al. 2009; McNulty et al. 2011). In humans, although lactococci are not a frequent element of intestinal microbiota, their presence in faeces was detected in several studies (Finegold et al. 1983; Millette et al. 2007; Lakshminarayanan et al. 2013; David et al. 2014).

The *L. lactis* subsp. *cremoris* IBB477 strain, originally isolated from raw cow milk in Poland, was demonstrated to possess adhesive properties towards bare and PGM-coated polystyrene at the single-cell level, by atomic force microscopy (AFM) (Le et al. 2011), as well as at the bacterial population level (Le et al. 2012; Radziwill-Bienkowska et al. 2014), and recently under dynamic conditions, using a shear stress flow chamber (Radziwill-Bienkowska et al. 2016). Owing to its adhesive properties, IBB477 (called for the purpose of the cited publication *L. lactis* subsp. *cremoris* IBB SC1) was used as a potential probiotic strain and was shown to survive in the chicken guts throughout their lifespan (42 days) (Sławińska et al. 2014). Furthermore, the IBB477 was selected as a candidate strain for development of an oral protective vaccine against avian influenza virus infections (Radziwill-Bienkowska et al. 2014). As we are interested in mechanisms of adhesion with special respect to the *Lactococcus* genus, our studies focus on the identification of molecular factors involved in adhesion of *L. lactis* subsp. *cremoris* IBB477 to the intestinal mucosa. Based on genomic analysis, putative adhesins of IBB477 strain were identified and the role in adhesion of one of the chromosomal genes (AJ89_07570) was confirmed (Radziwill-Bienkowska et al. 2016). In the present study, contribution of plasmid-encoded factors to adhesive properties of IBB477 strain was investigated. Plasmid-cured derivatives as well as deletion mutants were obtained and analysed. Adhesion tests were performed using non-coated and coated polystyrene plates and the mucus-secreting HT29-MTX cell line. Interactions between bacteria and mucus secreted by HT29-MTX cells were visualised by fluorescent staining and confocal microscopy. Finally, the resistance to digestive stress and the ability of IBB477 and selected deletion mutant to colonise the GIT of conventional C57Bl/6 mice were determined.

## Materials and methods

### Bacterial strains and growth conditions

Bacterial strains used in this study are listed in Table 1. The *L. lactis* IBB477 strain originates from the samples of raw cow milk collected in Poland at the turn of the century (Zycka-Krzesinska et al. 2015). Bacterial strains were generally cultured and stored as previously described (Radziwill-Bienkowska et al. 2016). For the adhesion test to HT29-MTX cells and the digestive stress test, bacterial strains were grown at 37 °C. In experiments with HT29-MTX cells, the strains were incubated in cell culture medium with reduced foetal bovine serum (FBS) [2% (*v*/*v*)]. For construction of deletion mutants, the growth medium was supplemented with erythromycin (Em; 100 μg ml^−1^ for *E. coli* and 5 μg ml^−1^ for *L. lactis*) and specific temperatures were applied as described previously (Radziwill-Bienkowska et al. 2016). In in vivo studies, bacteria were counted by plating mice faeces on M17-glucose (0.5% *w*/*v*; M17; Oxoid Ltd., Basingstoke, Hampshire, UK) supplemented with nalidixic acid (Nal; 40 μg ml^−1^) and Tet (100 μg ml^−1^—concentration necessary for elimination of Tet^r^ *Enterococcus* spp. present in faeces and permissive for the IBB477 strain) at 30 °C.

**Table 1:** Bacterial strains used in this study

Strain^a^	Relevant characteristic(s)	Source and/or reference
*E. coli*		
TG1	Δ(*hsdMS-mcrB*)5 Δ(*lac-proAB*) *supE thi1*F’(*traD36 proAB* ^*+*^ *lacI* ^*q*^ *ZΔM15*)	Carter et al. (1985)
EC1000	Km^r^, RepA^+^ MC1000, carrying a single copy of the pWV01 *repA* gene in the *glgB* gene	Leenhouts et al. (1991)
*L. lactis* subsp. *lactis*		
IL1403	Plasmid-free wild-type, low-adhesive control strain	INRA, Jouy-en-Josas, France (Chopin et al. 1984)
*L. lactis* subsp. *cremoris*		
MG1820	MG1363 carrying plasmid pMG820, low-adhesive control strain	LISBP, Université de Toulouse, CNRS, INRA, INSA, Toulouse, France (Maeda and Gasson 1986)
IBB477	Wild-type strain, Tc^r^	IBB PAS, Warsaw, Poland
IBB3171 (-a)	IBB477-derivative, -pIBB477a, Tc^s^	This study
IBB3172 (-b)	IBB477-derivative, -pIBB477b, Tc^r^	This study
IBB3176 (-c)	IBB477-derivative, -pIBB477c, Tc^r^	This study
IBB3178 (-d)	IBB477-derivative, -pIBB477d, Tc^r^	This study
IBB3173 (-ab)	IBB477-derivative, -pIBB477a, -pIBB477b, Tc^s^	This study
IBB3177 (-ac)	IBB477-derivative, -pIBB477a, -pIBB477c, Tc^s^	This study
IBB3177 (-ad)	IBB477-derivative, -pIBB477a, -pIBB477d, Tc^s^	This study
IBB3174 (-bc)	IBB477-derivative, -pIBB477b, -pIBB477c, Tc^r^	This study
IBB3175 (-abc)	IBB477-derivative, -pIBB477a, -pIBB477b, -pIBB477c, Tc^s^	This study
IBB3189 (Δ14140)	IBB477ΔAJ89_14140, Tc^r^	This study
IBB3190 (ΔprtP)	IBB477ΔAJ89_14230, Tc^r^	This study

^a^The name of strain used for the purpose of this study is given in brackets. Strains obtained in this study are deposited in the publicly accessible IBB PAS laboratory culture collection. Strain IBB477 is deposited in the Polish Collection of Microorganisms—PCM culture collection no. 2853

### Plasmid-curing method

The plasmid-free derivatives obtained in this study are presented in Table 1. Plasmid curing was performed by growth passages. A 10^3^-fold dilution of the saturated culture of IBB477 or its plasmid-free derivatives in M17-glucose medium with Tet was incubated for 72 h at 30 °C and used for inoculation of the subsequent passage. Appropriate dilutions of selected passages were plated on M17-glucose medium with Tet or in the absence of Tet in the case of plasmid pIBB477a curing, for which tetracycline-sensitive colonies were isolated on M17-glucose medium without antibiotic selection and compared with replicates on plates with Tet. The presence of other plasmids was analysed using multiplex colony PCR with primer pairs specific for each plasmid (Table 2). For all unique derivatives with different plasmid content, the absence of a single plasmid was verified using PCR with another primer pair (Table 2). In addition, to compare plasmid profiles, plasmid DNA was isolated using the Plasmid Midi AX purification kit (A&A Biotechnology, Gdynia, Poland) preceded by incubation in TES-lysozyme (10 mg ml^−1^) for 40 min at 43 °C. Plasmid DNA was then digested with *Eco*RI or *Hin*dIII restriction enzymes according to the manufacturer’s instructions (Fermentas, St. Leon-Rot, Germany). DNA was analysed by electrophoresis on agarose gels, which were subsequently photographed under UV light.

**Table 2:** Primer pairs used for the analysis of plasmid-free derivatives of IBB477 and for construction of IBB477 deletion mutants

Plasmid/mutant name	Primers^a^
pIBB477a	p477a_F TGCAAATCCTACACATGACACAAT p477a_R GCTTCAACGGCTTCTCCTAA
pIBB477b	p477b1_F GCGGAGCCAAGAGAAGGTA^b^ p477b1_R CTTAAAAGCATCAAACAAACT
	p477b2_F CAGCCAAGTAATCGTCGCATAA p477b2_R CGAATCCATCAAAGTTTAGGGTAT
pIBB477c	p477c1_F GGCAAACAATCCTGAAAAGTA^b^ p477c1_R GACATCAGCTTGCCCTACTCG
	p477c2_F TTGGAGATTATCGCTGGTGAACTA p477c2_R TCTAACCGCCAAACAACGAT
pIBB477d	p477d1_F TTATGACAGGGAGGCGTTAG^b^ p477d1_R CCGACCAATCGATAGCATAG
	p477d2_F CGCAGGAAGAAGTCCAAACC p477d2_R AGATACCTGCACGCTGTGTC
pIBB477e	p477e_F TCCGCTATGTCCATAATCCG^b^ p477e_R ATTTACGCCACCACTCTAGG
Δ14140	14140-u_F AACCTCTATCGCTCCCTATG 14140-u_R **CGAATT-C**AATGGTCACTTCCTGATTAGC (*Eco*RI) 14140-d_F **CGAA-TTC**ACGCTTCCCAATTAGTCAAC (*Eco*RI) 14140-d_R GGCCATCTTGATTGTTAGGG
ΔprtP	prtP-u_F AACAGTCACATTGGCGAAAG prtP-u_R C**GAA**-**TTC**AGCGGAAGCAACTGTGG (*Eco*RI) prtP-d_F C**GAATT**-**C**CGACATTGCTGACACATTG (*Eco*RI) prtP-d_R GATACGCTGCTGCCCTAAAC

^a^Restriction sites are indicated in bold letters

^b^Primer pairs used for multiplex PCR

### Construction of deletion mutants

Mutants were created using an integration-excision system based on the thermosensitive plasmid pGhost9 (Maguin et al. 1996) with added 3′ terminal thymidine to both ends after *Eco*RV digestion, as previously described (Radziwill-Bienkowska et al. 2016). The mutants and primer pairs used for their construction are presented in Tables 1 and 2, respectively.

### Adhesion tests to polystyrene, mucin and fibronectin

Adhesive properties of bacterial cells were tested on bare 96-well polystyrene microtiter plates (cat. no. 167008, Thermo Fisher Scientific, Nunc A/S, Waltham, MA, USA) as well as microtiter plates coated with type III mucin from porcine stomach (PGM) (cat. no. M1778, Sigma-Aldrich, St. Louis, MO, USA) [10 mg ml^−1^] or fibronectin from human plasma (FN) (cat. no. F2006, Sigma-Aldrich, St. Louis, MO, USA) [20 μg ml^−1^] dissolved in phosphate-buffered saline (PBS), pH = 7.4 (BioShop Canada Inc., Burlington, Ontario, Canada), using the technique described for the IBB477 strain and its deletion mutants in chromosomal genes (Radziwill-Bienkowska et al. 2016). Briefly, bacterial suspensions (OD_600 nm_ = 1) were incubated in bare (polystyrene (PS)) or coated plates (PS + PGM, PS + FN) for 3 h at 30 °C, unbound bacteria were washed away with water, and adherent bacteria were stained with crystal violet. Each microtiter plate included the control strains: the wild-type IBB477 strain, which is highly adhesive to PS, PS + PGM and PS + FN (Le et al. 2011, 2012; Radziwill-Bienkowska et al. 2014, 2016), *L. lactis* MG1820, which has low adhesion to PS and PS + PGM (Dague et al. 2010; Le et al. 2011, 2012; Radziwill-Bienkowska et al. 2016), and *L. lactis* IL1403, which has low adhesion to PS, PS + PGM and PS + FN (Radziwill-Bienkowska et al. 2016), as well as blank wells with PBS. The adhesion was expressed as the optical density (OD_583 nm_) of stained cells. The average value of at least six measurements from three independent experiments was calculated after rejecting extreme results.

### Cell line and culture conditions

The HT29-MTX mucus-secreting subpopulation of human colon carcinoma cell line HT29 was kindly provided to Muriel Thomas (Micalis, INRA, Jouy-en-Josas, France) by Dr. Thécla Lesuffleur (INSERM UMR S 938, Paris, France) (Lesuffleur et al. 1990). The HT29-MTX cells were routinely grown in Dulbecco’s modified Eagle’s minimal essential medium (DMEM) containing phenol red and 4.5 g l^−1^ glucose (Lonza, Verviers, Belgium), supplemented with: 10% (*v*/*v*) heat-inactivated FBS (Lonza, Verviers, Belgium), 1% (*v*/*v*) l-glutamine 200 mM (Lonza, Verviers, Belgium) and 1% (*v*/*v*) penicillin-streptomycin mixture (10,000 U ml^−1^ and 10,000 μg ml^−1^, respectively) (Lonza, Verviers, Belgium). The cells were seeded at a concentration of 2.5 × 10^4^ cells cm^−2^ in six-well tissue culture plates (Thermo Fisher Scientific, Nunc A/S, Waltham, MA USA) or on glass coverslips placed in 24-well tissue culture plates (Thermo Fisher Scientific, Nunc A/S, Waltham, MA USA) for adhesion experiments and for fluorescent staining experiments, respectively. Two days before the experiments, antibiotics were no longer added to the cell culture medium. HT29-MTX cells were used between passages 27 and 46. To ensure full differentiation of cells, experiments were carried out 20 to 22 days post seeding. The cells were maintained at 37 °C in a humidified atmosphere with 10% CO_2_, and the culture medium was changed daily.

### Adhesion test to HT29-MTX cells

Adhesion experiments to the HT29-MTX cell line were performed according to the method described previously by Turpin et al. (2012), with some modifications. Bacterial cells from 12-h precultures were used to inoculate cell culture medium with reduced FBS concentration [2% (*v*/*v*)] at a starting OD_600 nm_ = 0.05. After an 8-h adaptation phase, bacteria were washed, suspended in fresh culture medium with 2% FBS and diluted to obtain bacterial cell concentration of 3–4 × 10^7^ CFU ml^−1^. HT29-MTX cells were gently washed twice with PBS, pH = 7.5 (Lonza, Verviers, Belgium), and 2 ml of bacterial suspensions was added to each well, resulting in bacterial cell-to-epithelial cell ratio (MOI) of 10:1. The bacterial and epithelial cells were co-incubated for 2 h at 37 °C in a humidified atmosphere with 10% CO_2_. After incubation, HT29-MTX cells were washed twice with PBS to remove unbound bacteria, scraped with 0.1% (*v*/*v*) Triton X-100 (Sigma-Aldrich, St. Louis, MO, USA), passed five times through a 21-gauge needle and incubated for 30 min at room temperature. Using the plating method, the number of viable bacterial cells was determined in the cell pellet (adherent bacterial cells) and in the bacterial suspension after incubation in empty wells (control input). Results were expressed as the percentage of adherent bacterial cells with respect to the amount of bacteria added (control input). At least three independent experiments were performed, and for each well, at least three serial dilutions in duplicate were carried out.

### Fluorescent staining of mucus secreted by HT29-MTX cells and adherent bacteria

The adherent bacteria and mucus secreted by HT29-MTX cells were stained after performing an adhesion test. Bacterial suspensions were prepared and co-incubated with HT29-MTX cells as described earlier. The amount of bacteria added to each well was adjusted to 0.5 ml, as for staining purposes, HT29-MTX cells were grown on glass coverslips placed in 24-well tissue culture plates. After co-incubation, HT29-MTX cells were washed twice with PBS, pH = 7.5 (Lonza, Verviers, Belgium), to remove unbound bacteria, fixed with 4% paraformaldehyde (PFA) (Thermo Fisher Scientific, Fisher Scientific, Waltham, MA USA) for 15 min at room temperature and washed three times with cold PBS. First, the adherent bacteria were stained using fluorescent in situ hybridisation (FISH), by the technique adapted from Rochet et al. (2004), with EUB 338 probe (5′-GCTGCCTCCCGTAGGAGT-3′) specific for the domain *Bacteria* (Amann et al. 1990) and labelled with fluorescein isothiocyanate (FITC) (Qbiogene, Evry, France). Glass coverslips with fixed cells were incubated with 50 μl of hybridisation solution (900 mM NaCl; 20 mM Tris–HCl, pH 8.0; 0.01% sodium dodecyl sulphate (SDS); 30% formamide) containing 6 ng μl^−1^ of fluorescent probe for 16 h at 35 °C in humidified atmosphere. Then, the coverslips were washed three times for 10 min at 37 °C with washing solution (64 mM NaCl; 20 mM Tris–HCl, pH 8.0; 0.01% SDS) and three times with cold PBS. Next, immunofluorescence staining of mucus produced by HT29-MTX cells was performed using the anti-MUC5AC antibody as MUC5AC is the major gel-forming mucin in this in vitro model (Lesuffleur et al. 1993). Glass coverslips after FISH staining were blocked for 1 h at room temperature with 3% (*w*/*v*) bovine serum albumin (BSA) (Sigma-Aldrich, St. Louis, MO, USA) in PBS. Then, coverslips were incubated in a 1:100 dilution of rabbit anti-MUC5AC (H-160) primary antibody (Santa Cruz Biotechnology, Heidelberg, Germany) for 1 h at room temperature. Indirect fluorescence was carried out for 1 h at room temperature using 1:400 goat antirabbit antibody conjugated with Alexa Fluor 647 (Thermo Fisher Scientific, Invitrogen, Waltham, MA USA). After each incubation with antibody, HT29-MTX cells were washed four times with cold PBS for 10 min. Stained glass coverslips were mounted on Superfrost microscope slides (Thermo Fisher Scientific, Thermo Scientific, Waltham, MA USA) using ProLong Gold antifade reagent (cat. no. P36930, Thermo Fisher Scientific, Life Technologies, Waltham, MA USA). For each bacterial strain, at least two independent experiments were performed in duplicate. Controls included no bacteria, no HT29-MTX cells and no primary antibody.

### Confocal microscopy

Stained HT29-MTX cells with adherent bacteria were examined using a motorised stage on the Leica TCS SP8 AOBS inverted confocal microscope (Leica Microsystems, Mannheim, Germany) at the INRA MIMA2 platform (http://www.jouy.inra.fr/mima2). Observations were performed using ×63/1.40 oil immersion objective. FITC was excited at 488 nm using an argon laser [output power at 30%, acousto-optic tunable filter (AOTF) ca. 5%] and was detected between 500 and 550 nm. Simultaneously, Alexa Fluor 647 was excited at 633 nm using He/Ne laser (AOTF ca. 0.7%) and the emitted fluorescence was recorded from 638 to 790 nm. Signals were recorded with hybrid detectors (HyD) in standard mode with 100% digital gain. Single 3D acquisitions as well as mosaics (3 × 3, with automated merging and stitching of 10–20%) were acquired at a scan speed of 600 Hz in bidirectional mode with scanning zoom 1, an image resolution of 1024 × 1024 pixels, a line average of 2 and a *z*-step between each *xy* image for a *z*-stack of 0.3 μm. The images were analysed, and graphical representations were prepared using the IMARIS 7.7.2 software (Bitplane, Zurich, Switzerland).

### Digestive stress test

Resistance of bacterial strains to acid and bile salts was assessed using the method adapted from Kechaou et al. (2013). Bacteria from overnight cultures were washed twice with PBS, pH = 7.5 (Lonza, Verviers, Belgium), and diluted to OD_600 nm_ = 0.5 in 1 ml of PBS, pH = 7.5 (control); PBS, pH = 3 (acid stress); and PBS, pH = 7.5, containing 3 g l^−1^ of two bile salts: sodium cholate and sodium deoxycholate (Sigma-Aldrich, St. Louis, MO, USA) (bile salt stress). After incubation for 1 h at 37 °C, bacterial cells were washed with PBS and suspended in M17-glucose (0.5% *w*/*v*) medium. For each strain and each condition, four 2-fold serial dilutions were prepared in duplicate on a sterile 384-well microtiter plate (Greiner Bio-One GmbH, Frickenhausen, Germany) starting from OD_600 nm_ = 0.025. Growth of bacteria at 37 °C was monitored every 15 min for 28 h by measuring the OD_600 nm_ with the Infinite M200 Pro plate reader (Tecan, Maennedorf, Switzerland). The resistance of bacterial strains to digestive stress was determined by measuring the growth delay (i.e. delay in time needed to reach mid-exponential phase) between stressed and non-stressed cultures. For each strain and each condition, four growth delays corresponding to the four dilutions on the microtiter plate were averaged.

### In vivo approach

Conventional C57Bl/6 mice (males, 6 weeks of age; Janvier Labs, Le Genest Saint Isle, France) were maintained under normal husbandry conditions in the animal facilities of the INRA IERP Unit (Jouy-en-Josas, France). All animal experiments began after 1-week acclimation and were performed according to the European Community rules of animal care and with authorisation 12-164 of the French Veterinary Services. Overnight cultures of bacteria, performed in 200 ml of M17-glucose (0.5% *w*/*v*) medium supplemented with antibiotics (Nal and Tet), were washed three times with PBS, pH = 7.5 (Lonza, Verviers, Belgium) and suspended in 1.5 ml of PBS to a final concentration of OD_600 nm_ = 100. Mice (*n* = 6) were intragastrically administered with 100 μl of bacterial suspensions, i.e. 3 × 10^9^ CFU per mouse (verified by plating). In order to investigate the persistence of bacteria in the GIT, faeces were collected for plating just before the gavage (control) and after 4, 8, 48 and 72 h. During that time, the bedding was changed daily. To determine the localisation of bacteria in the GIT, 72 h after first gavage, mice were administered again with bacteria (2 × 10^9^ CFU per mouse) and sacrificed by cervical dislocation 24 h later. After sacrifice, contents of stomach, ileum, caecum and colon were collected for plating. Collected faeces and intestinal contents were suspended in PBS (100 μl per 10 mg), and for each sample, four serial dilutions were plated on appropriate selective plates. The authenticity of the counted colonies (one colony for each strain for each time point and each intestinal region) was verified by colony PCR with two primer sets: 68F (5′-GATGAAGATTGGTGCTTGCA-3′) and 1406R (5′-ACGGGCGGTGTGTRC-3′) specific for *L. lactis* subsp. *cremoris* (Salama et al. 1991) and Mub-outF (5′-TGCTTACGCTCATATCCTTC-3′) and Mub-outR (5′-CATTTGCTTGCTCAGAGTTC-3′) amplifying AJ89_12755 gene (*mub* gene), which was shown to be longer in the IBB477 strain than that found in other *L. lactis* subsp. *cremoris* genomes (Radziwill-Bienkowska et al. 2014).

### Statistical analysis

Statistical analysis was performed using GraphPad Prism software (GraphPad Software, Inc., San Diego, CA). Results from adhesion tests to mucin, fibronectin and HT29-MTX cells were expressed as means ± SD and analysed by Welch’s *t* test. Differences were considered statistically significant when *p* value was <0.05. In vivo data were presented as 25th to 75th percentile boxes with median line in the middle and all individual values marked.

## Results

### Influence of plasmids on *L. lactis* IBB477 adhesion to bare polystyrene

**Fig. 1:**
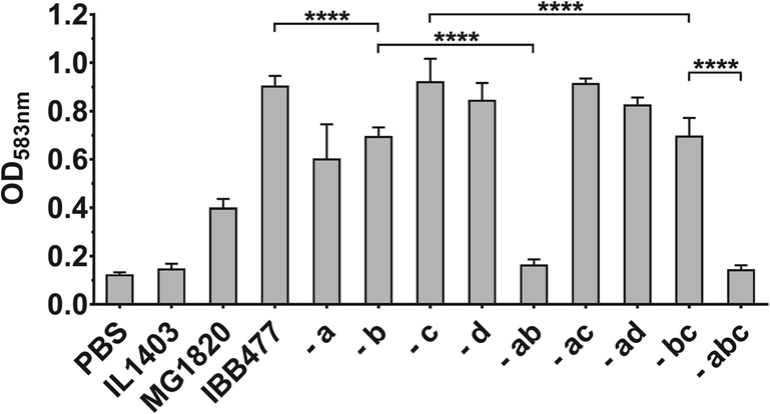
Adhesion of IBB477 plasmid-free derivatives to bare polystyrene (PS). Adhesion is expressed as optical density (OD_583 nm_) of stained cells. Means ± standard deviations from three independent experiments are shown. The *p* values were calculated using Welch’s *t* test (****p value <e^−4^)

To elucidate the role of plasmids in adhesion of *L. lactis* IBB477 strain, a set of plasmid-free derivatives was obtained using a plasmid-curing method based on passages and prolonged growth without any toxic compounds to minimise spontaneous mutations. The absence of plasmids in derivatives was confirmed by PCR analyses and by comparing plasmid profiles after agarose gel electrophoresis of purified plasmids and plasmid DNA digested with restriction enzymes (data not shown). The obtained derivatives include those lacking single plasmids: pIBB477a, pIBB477b, pIBB477c and pIBB477d; two plasmids: pIBB477a and b, pIBB477a and c, or pIBB477a and d, as well as one derivative lacking three plasmids pIBB477a, b and c (Table 1). The adhesive properties of IBB477 derivatives were analysed using adhesion tests on bare PS (Fig. 1). A significant decrease in adhesion to bare PS was observed for all derivatives without plasmid pIBB477b. A decrease in adhesion was also observed for derivative lacking single plasmid pIBB477a; however, results were less reproducible, and the effect was not visible for derivatives lacking two plasmids pIBB477a and c or pIBB477a and d. The lowest adhesion level among plasmid-free derivatives was measured for all derivatives lacking both plasmids pIBB477a and b. Derivatives without single plasmids pIBB477c or d had similar adhesion level as the wild-type IBB477 strain. These results suggest that one or more genes that are localised on plasmid pIBB477b play a role in adhesion of the IBB477 strain.

### Adhesion of deletion mutants in putative adhesion genes localised on plasmid pIBB477b to bare, mucin- and fibronectin-coated PS

**Fig. 2:**
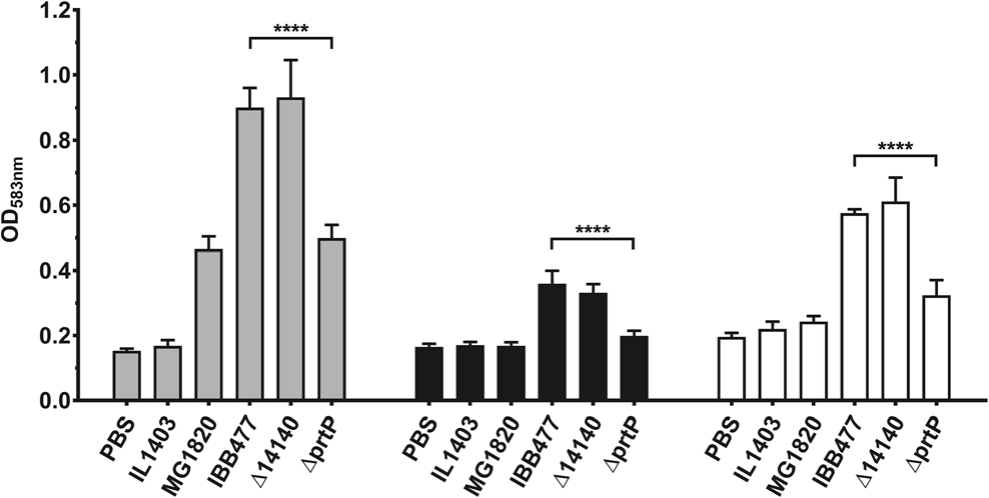
Adhesion of IBB477 and its two deletion mutants in putative adhesion genes localised on plasmid pIBB477b to bare polystyrene (PS) (*grey bars*), mucin-coated polystyrene (PS + PGM) (*black bars*) and fibronectin-coated polystyrene (PS + FN) (*white bars*). Adhesion is expressed as optical density (OD_583 nm_) of stained cells. Means ± standard deviations from three independent experiments are shown. The *p* values were calculated using Welch’s *t* test (****p value <e^−4^)

Based on our recently published results on IBB477 (Radziwill-Bienkowska et al. 2016), only two proteins encoded by genes located on the pIBB477b plasmid were found as putative adhesins according to their extracellular or cell wall localisation: the AJ89_14230 gene coding for peptidase S8 with homology to *L. lactis* PrtP protein and the gene with locus tag AJ89_14140 annotated as a hypothetical protein. These two genes were thus selected for deletion and functional analysis. The adhesive properties of IBB477 deletion mutants, ΔprtP and Δ14140, were tested on bare polystyrene plates (PS) as well as on plates coated with mucin (PS + PGM) and fibronectin (PS + FN) in comparison with the wild-type strain (Fig. 2). Adhesion was expressed as the optical density (OD_583 nm_) of stained cells. One of the IBB477 plasmidic deletion mutants (ΔprtP) in gene AJ89_14230 adhered significantly less than the wild-type IBB477 strain, with *p* value <e^−4^ (95% CI = −0.44 to −0.37), *p* value <e^−4^ (95% CI = −0.18 to −0.14) and *p* value <e^−4^ (95% CI = −0.28 to −0.22) to PS, PS + PGM and PS + FN, respectively. ΔprtP showed ca. 55% of adherence compared with IBB477 strain in all three cases. Deletion of AJ89_14140 gene did not significantly change the adhesion level of the corresponding deletion mutant to any of tested surfaces compared to the wild-type strain. Despite many efforts to construct the strain complementing ΔprtP, all attempts failed, probably owing to the length (more than 6 kb) of the nucleotide sequence of the AJ89_14230 gene. However, in our study, several independently constructed deletion mutants in this gene located on pIBB477b plasmid were analysed and presented similar levels of adhesion to each tested surface (data not shown).

### Adhesion to the mucus-secreting cell line of the IBB477 strain and its deletion mutant (ΔprtP)

**Fig. 3:**
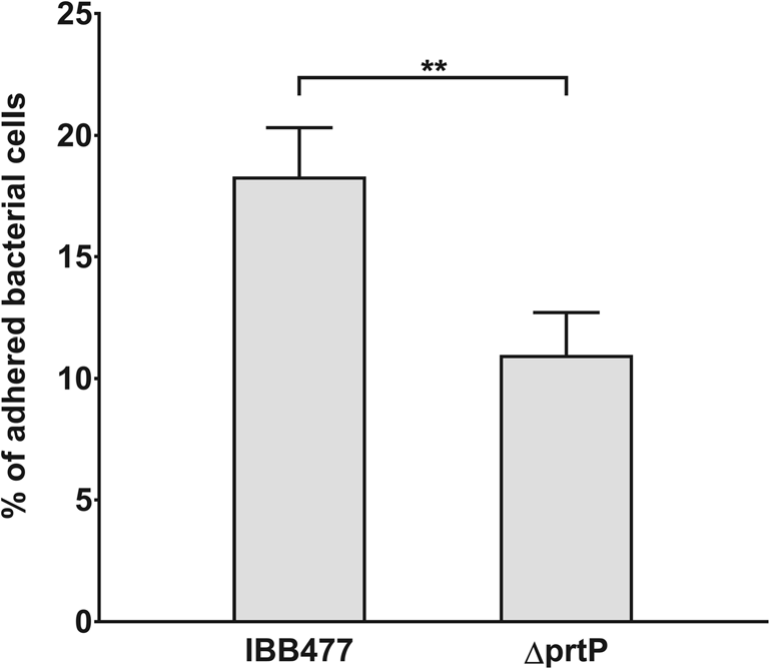
Adhesion of IBB477 and its deletion mutant in AJ89_14230 gene (ΔprtP) to mucus-secreting HT29-MTX cells. Adhesion is expressed as percentage of adherent bacterial cells with respect to the amount of bacteria added. Means ± standard deviations from three independent experiments are shown. The *p* values were calculated using Welch’s *t* test (**p value <e^−2^)

To further investigate the role of the AJ89_14230 gene in adhesive behaviour of IBB477 strain, adhesion test to the mucus-secreting HT29-MTX cell line was performed for the wild-type IBB477 vs. its ΔprtP mutant. The adaptation phase of bacteria in cell culture medium was set for 8 h as the maximal OD_600 nm_ for both strains was observed for 7.5 h and as after 9 h of incubation, OD_600 nm_ and number of viable cells began to decrease (data not shown). Susceptibility of tested strains to 0.1% (*v*/*v*) Triton X-100 was verified, and no significant differences in survival rates were observed in comparison with non-treated bacteria (data not shown). Based on CFU counting, the percentage of adherent bacterial cells to HT29-MTX cells was determined for IBB477 strain and its deletion mutant (ΔprtP) (Fig. 3). The level of adhesion obtained for IBB477 (18%) was significantly higher than for ΔprtP (11%) with *p* value <e^−2^ (95% CI = 3.87 to 10.8).

**Fig. 4:**
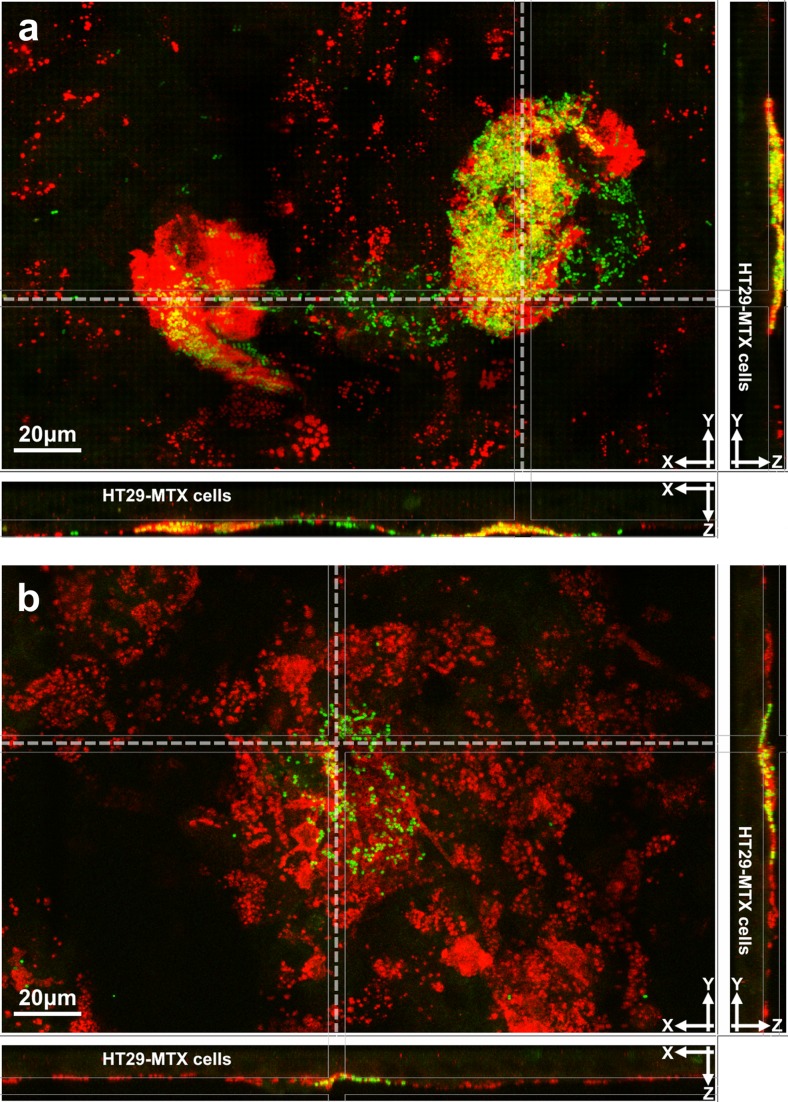
Visualisation of interactions between bacteria (in *green*, FISH probe Eub338) and mucus (in *red*, anti-MUC5AC antibody) secreted by HT29-MTX cells with confocal microscopy. Representative confocal *z*-stack images of HT29-MTX cells, mucus and bacterial strains: IBB477 (**a**) and its ΔprtP mutant (**b**) after 2-h adhesion are displayed in easy 3D section view with large z-sections (*x*, *y*) at the level of mucus and orthogonal *x* (*y*, *z*) and *y* (*x*, *z*) sections on the right and on the bottom, respectively. *Yellow colour* indicates bacteria that are embedded in mucus. Positions of orthogonal sections are indicated on z-sections with *dashed lines*. Localisation of HT29-MTX cells (slight autofluorescence in *green*) is marked on orthogonal sections. For all sections, the extended view with signal gathered from thickness of 5 μm was selected. The images were prepared using the IMARIS software (Color figure online)

To visualise the interactions between bacteria and HT29-MTX cells, stained cells and bacteria were examined under confocal microscope for IBB477 and its deletion mutant (ΔprtP). Representative images are shown in Fig. 4a, b. First, mucus stained with MUC5AC antibody did not form a uniform layer over the cell surface, but rather a patchy structure (Fig. 4a, b), and the maximal thickness of patches was 5 μm. Then, for both strains, it was observed that, in general, bacterial cells formed clusters that were embedded in mucus and sparsely distributed along the sample. The density of bacterial clusters was higher for IBB477 strain than for ΔprtP mutant. It was noted that for both strains, some bacterial cells were not localised in mucus but rather in direct contact with the apical side of HT29-MTX cells.

### Transit of the IBB477 strain and its deletion mutant (ΔprtP) in conventional C57Bl/6 mice

In order to investigate the ability to persist in the GIT of conventional rodents and observe the potential differences in the transit between IBB477 and its deletion mutant (ΔprtP), in relation to their different in vitro adhesive phenotypes, a C57Bl/6 mice model was used.

As a first step, an in vitro digestive stress test was performed to verify whether deletion of AJ89_14230 gene did not change the resistance properties of the ΔprtP mutant in comparison with the wild-type IBB477 strain. Bacteria were subjected to acid (pH = 3) and bile salts to mimic the passage through the GIT. Data were then analysed according to Kechaou et al. (2013). No significant differences in the growth delays between tested strains were observed (acid stress 5.9 h ± 0.6 vs. 6.5 h ± 0.7 and bile salt stress 8.5 h ± 1.3 vs. 8.5 h ± 1.6 for IBB477 vs. ΔprtP), indicating similar levels of resistance to digestive stresses for both strains.

**Fig. 5:**
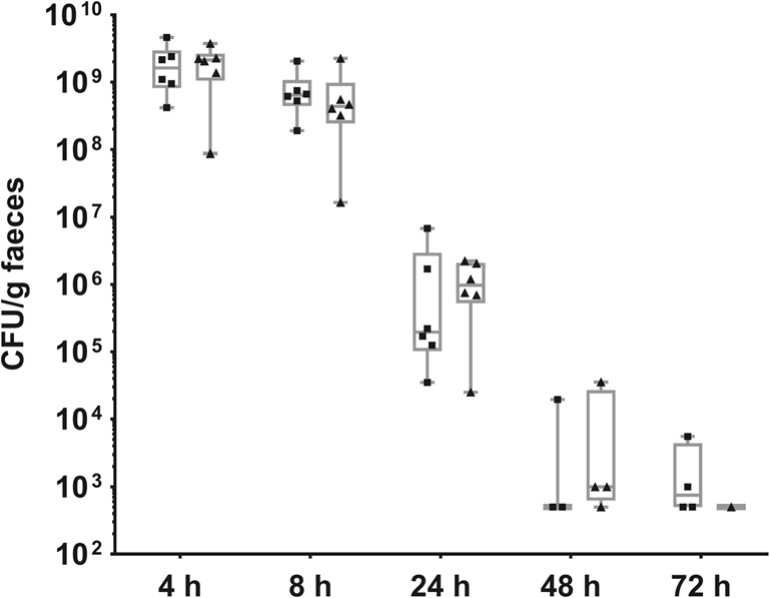
Persistence of IBB477 (*squares*) and its deletion mutant in AJ89_14230 gene (ΔprtP) (*triangles*) in the GIT of conventional C57Bl/6 mice after single oral administration. Bacterial counts in faeces of mice (*n* = 6) are presented as 25th to 75th percentile *boxes* with *median line* in the *middle* and all individual values marked

Next, persistence of IBB477 and its deletion mutant (ΔprtP) in the GIT of conventional C57Bl/6 mice after single oral administration was determined (Fig. 5). For both strains, the number of viable bacterial cells in faeces of mice progressively decreased between 4 and 48 h after gavage (from ca. 10^9^ to 10^4^ CFU/g faeces). At 72 h after administration, IBB477 was still detected at 10^3^ CFU/g faeces in four mice, but ΔprtP was detected in the faeces of only one animal (*n* = 6 mice in both cases).

**Fig. 6:**
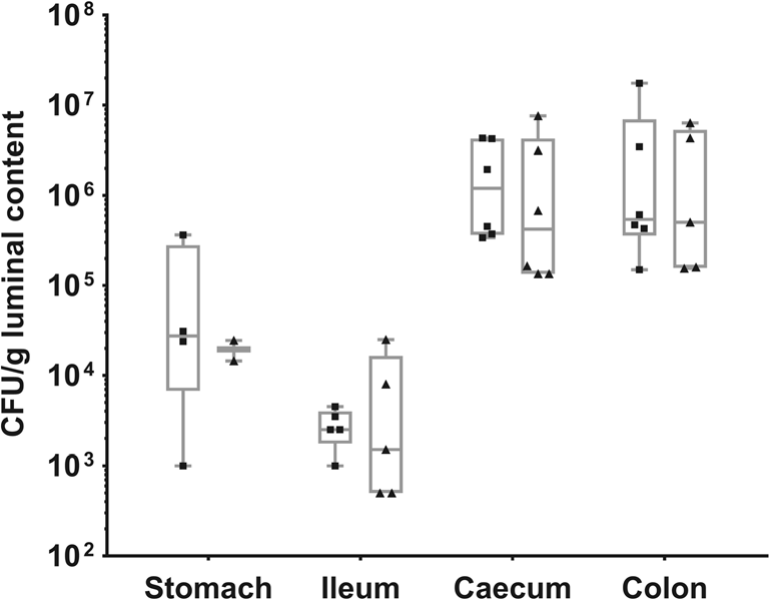
Amount of IBB477 (*squares*) and its deletion mutant in AJ89_14230 gene (ΔprtP) (*triangles*) in the GIT (stomach, ileum, caecum and colon) of conventional C57Bl/6 mice 24 h after gavage. Bacterial counts in luminal contents of mice (*n* = 6) are presented as 25th to 75th percentile *boxes* with *median line* in the *middle* and all individual values marked

Finally, the amounts of IBB477 strain and its ΔprtP mutant in different parts of the GIT of conventional C57Bl/6 mice 24 h after gavage were investigated (Fig. 6). Both strains were detected in all intestinal regions at similar levels with the highest amount found in the colon and caecum (ca. 10^6^ CFU/g luminal content).

## Discussion

In the present work, adhesion and persistence of *L. lactis* subsp. *cremoris* IBB477 strain were analysed by in vitro and in vivo approaches, respectively. In our previous study on adhesion of IBB477, we demonstrated that the chromosomal gene encoding the AJ89_07570 protein containing DUF285, C-term_anchor (recently reclassified as MucBP (PF06458) domain) and four Big_3 domains is involved in adhesion to abiotic surfaces as well as mucins (Radziwill-Bienkowska et al. 2016). Here, we focus on the role of molecular factors localised on plasmids in the adhesive properties of the IBB477 strain and their impact on its persistence in the GIT.

Using a plasmid-curing method, a set of plasmid-free derivatives of IBB477 was obtained and analysed. The frequency of loss of plasmids differed for each plasmid (data not shown). Adhesion tests on bare PS plates revealed that two plasmids, pIBB477a and b, are involved in adhesion of the IBB477 strain. Whereas for all obtained derivatives without plasmid pIBB477b (-b, -ab, -bc and -abc), the level of adhesion was lower than that for the wild-type IBB477 strain, the role of plasmid pIBB477a in adhesion is less straightforward. For derivatives that only lost pIBB477a plasmid, adhesion was lowered but the variation level was high. For derivatives without two plasmids -ac and -ad, no significant changes were observed. However, the simultaneous loss of pIBB477a and pIBB477b plasmids (-ab and -abc derivatives) resulted in a very pronounced loss of adhesive properties of IBB477 strain to bare PS (similar level of adhesion as for IL1403 control strain). This observation strongly suggests that there is an interaction between molecular factors encoded by both plasmids that plays a role in adhesion of the IBB477 strain. Further analysis is required to confirm this hypothesis. A similar hypothesis claiming that the open reading frames carried by different plasmids or located on plasmids and on the chromosome in lactococci form functional biological systems have been proposed (Górecki et al. 2011). Notably, the role of multiple cell surface proteins in adhesive properties was previously reported for other LAB strains such as *Lactobacillus acidophilus* NCFM and *Lactobacillus gasseri* SBT2055 (Buck et al. 2005; Arai et al. 2016). In the present study, we decided to focus on further investigation of the role of plasmid IBB477b, owing to its major role in the adhesive phenotype of the IBB477 strain.

On the recently published list of putative adhesins of IBB477 strain (Radziwill-Bienkowska et al. 2016), there are only two proteins encoded by genes localised on plasmid pIBB477b, i.e. the hypothetical protein of extracellular localisation (AJ89_14140) and the cell wall-attached peptidase S8 (AJ89_14230), which is homologous to the *L. lactis* PrtP protein. Deletion mutants in corresponding genes were created and analysed in terms of adhesion to bare microtiter plates or plates coated with mucin or fibronectin. AJ89_14230 protein was found to mediate adhesion to bare, mucin- and fibronectin-coated PS. Cell surface-associated macromolecules are considered to play an important role in the adhesion of LAB to the GIT (Sengupta et al. 2013). Notably, AJ89_14230 protein of IBB477 has high sequence similarity to proteinase PrtP from the *L. lactis* subsp. *cremoris* Wg2 strain (EMBL accession number M24767; Blastp result 100% query coverage and 94% identity), which was shown to enhance cell surface hydrophobicity and mediate adhesion to solid surfaces such as glass, polytetrafluorethylene (PTFE) and octadecanethiol (ODT) self-assembled monolayer (SAM) when introduced in the *L. lactis* subsp. *cremoris* MG1363 strain (Habimana et al. 2007; Bulard et al. 2012).

The IBB477 strain and its deletion mutant in the AJ89_14230 gene (ΔprtP) were further investigated in vitro using the mucus-secreting HT29-MTX cell line. As expected, the percentage of adherent bacterial cells was significantly lower for ΔprtP than for IBB477. Confocal images showed that both tested strains, in general, formed cell clusters embedded in mucus. However, the density of those clusters varied depending on the strain (i.e. a higher amount of aggregated bacterial cells was observed for IBB477 than for ΔprtP mutant). Dense clusters of bacteria interacting with mucus secreted by HT29-MTX cells have previously been observed by scanning electron microscopy (SEM) for adhering *Lb. acidophilus* BG2FO4 strain (Coconnier et al. 1992). It was also reported that *Helicobacter pylori* forms discrete foci or clusters throughout the mucus layer and close to the epithelium of a subpopulation of HT29-MTX cells (HT29-MTX-E12 cell line), when investigated by confocal microscopy (Dolan et al. 2012). Therefore, the ability to form dense clusters in mucus might be a characteristic feature of strains that are capable of colonising the mucus layer. In this study, we also observed that some of the bacterial cells were in direct contact with the apical side of HT29-MTX cells. It is possible that they were interacting with membrane-associated mucins, most probably MUC1 or MUC3, as these two transmembrane mucins were found to be expressed at high levels in differentiated HT29-MTX cells (Lesuffleur et al. 1993). Notably, in our experiment, mucus secreted by HT29-MTX cells did not form a uniform layer over the cell surface but rather a patchy structure with thickness of up to 5 μm. This observation is in agreement with previous studies in which mucus patches were visualised in this in vitro model (Coïc et al. 2012; Sperandio et al. 2013; Etzold et al. 2014). However, it was also reported that mucus secreted by HT29-MTX cells formed a dense gel layer entirely covering the cell surface (Coconnier et al. 1992; Gouyer et al. 2001; Gibbins et al. 2015). These discrepancies stress the necessity of mucus examination when HT29-MTX cells are used for adhesive experiments.

To investigate the ability of IBB477 strain and its ΔprtP mutant to persist in the GIT colonised with indigenous microbiota, conventional C57Bl/6 mice were used. Both the wild-type strain and its mutant were detected in faeces of mice up to 72 h. Although at this time point, IBB477 was still present in the faeces of four mice and ΔprtP only in one (out of six), the time of transition was too short to detect statistically significant differences in their persistence. The possible explanation for lack of colonisation is that the IBB477 strain is not able to overcome the barrier that is created by the indigenous microbiota present in the GIT of C57Bl/6 mice. In fact, it has been previously shown that the colonisation resistance of the resident intestinal microbiota of conventional animals often results in the elimination of introduced bacteria (van der Waaij et al. 1971; Zhang et al. 2016). Notably, persistence of some other *L. lactis* subsp*. cremoris* strains in the gut of conventional rodents was lower than for IBB477 as they were no longer detected in the faeces of animals 24–48 h after last administration (Schlundt et al. 1994; Brockmann et al. 1996; Watson et al. 2008). This may be related, at least to some extent, to the improved adhesive properties of IBB477 strain. Indeed, it was recently shown that the longer persistence of *L. lactis* CNCM I-1631 strain in conventionalised rats was partly imputable to its adherence ability to the host mucosa (Zhang et al. 2016). In addition, Watson et al. (2008) demonstrated that introduction of *Listeria monocytogenes* bile resistance mechanism in *L. lactis* NZ9000 resulted in improvement of its persistence from 24 to 72 h. In combination with adhesive properties, other phenotypical traits could thus be involved, the utilisation of mucin-derived carbon sources, e.g. *N-*acetylglucosamine and mannose (Roy et al. 2008), being another adaptation strategy for *L. lactis.*

Apart from temporal, spatial transit of IBB477 and its ΔprtP mutant in the GIT of conventional C57Bl/6 mice was also determined. The highest amount of bacteria 24 h after gavage, ca. 10^6^ CFU/g luminal content, was observed in the caecum and colon. Such levels were consistent with values obtained during the kinetics study in faeces 24 h after gavage. Relatively high amounts of bacteria were also detected in the stomach contents of approximately half of the mice, likely due to the fact that mice are coprophages. Similar results were obtained for the *L. lactis* WH-C1 strain 26 h after administration to Swiss Albino mice (Wang et al. 2011). The same concentrations of bacteria as those observed in our study were present in the caecum and stomach, whereas for the ileum, the amount of WH-C1 cells was around ten times higher. Notably, as for the WH-C1 strain, IBB477 was still present in the GIT of mice 3 days after administration, albeit at a lower amount (10^3^ CFU g^−1^ for IBB477 and 10^4^ CFU g^−1^ for WH-C1). The differences in the levels of remaining bacteria as well as bacterial cells present in the ileum can result from specific characteristics of tested strains and/or different mouse models.

Our results suggest that LAB strains expressing peptidase with homology to *L. lactis* PrtP protein could possess the increased adhesive properties to the intestinal mucosa. Adhesive properties can potentially prolong the contact between exogenously applied bacteria and the host, and therefore enhance the desired beneficial effect. Previous studies showed that some lactococcal strains were either able to colonise the human GIT in subdetectable levels or able to transiently colonise the gut when introduced with food (Finegold et al. 1983; Millette et al. 2007; Lakshminarayanan et al. 2013; David et al. 2014). Notably, there is increasing evidence that food-borne bacteria are biologically active in the colon, and therefore, they might contribute to the functions of gut microbiota (Zhang et al. 2016). Even though the IBB477 strain is not able to colonise the GIT of conventional C57BL/6 mice, it was shown to present the adhesive properties towards mucins as well as mucus-secreting cell line. Regarding such strains that are able to bind to mucus and are transiently present in the GIT, their use as functional foods (probiotics) may require the long-term consumption.

Overall, we demonstrated that cell wall-associated peptidase S8 (PrtP), encoded by the AJ89_14230 gene localised on plasmid pIBB477b, is a key factor in adhesion of *L. lactis* IBB477 strain, rather than conferring it a selective advantage in the gut of conventional C57BL/6 mice. With regard to our previous results (Radziwill-Bienkowska et al. 2016), we conclude that both chromosomal and plasmid-encoded molecular factors contribute to adhesive properties of *L. lactis* IBB477.
